# Live birth in woman with premature ovarian insufficiency and 46, XY karyotype after chemotherapy and bone marrow transplant: a case report

**DOI:** 10.1186/s12884-023-05464-1

**Published:** 2023-03-13

**Authors:** Yaojia Zhang, Haiyan Wang, Xiaoqin Pan

**Affiliations:** Department of Assisted Reproduction Center, Huzhou Maternity and Child Health Care Hospital, Huzhou, China

**Keywords:** Premature ovarian insufficiency, Disorders of sex development, Bone marrow transplant, Live birth, Case report

## Abstract

**Background:**

Premature ovarian insufficiency (POI) is a clinical syndrome defined by loss of ovarian function before the age of 40 years, characterized by elevated serum gonadotropin levels and decreased estrogen levels with menstrual disturbance. POI can be natural or iatrogenic such as after chemotherapy, radiotherapy and surgery.

**Case presentation:**

In this study, we describe a successful live birth in a 31-year-old woman with POI and 46, XY Karyotype after being treated with chemotherapy and bone marrow transplant (BMT) for acute non-lymphocytic leukemia when she was 17 years old. With amenorrhea or oligomenorrhea for 11 years, her serum level of FSH was up to 35.0 IU/L and 53.0 IU/L taken 4 weeks apart, which can be diagnosed as POI. After controlled ovarian stimulation treatment for three cycles with different protocols and frozen-thawed embryo transfer (FET), she finally got a successful pregnancy and had a live birth later.

**Conclusions:**

This case report serves as a reminder that karyotype of peripheral blood may mislead the diagnosis as disorders of sex development (DSD). It also demonstrates that it is possible for a woman with chemotherapy and bone marrow transplant induced POI can have successful pregnancy and live birth with appropriate therapy. Furthermore, as age may plays a predominant role in fertility rather than residual ovarian reserve, active treatment may be concerned for women with POI at younger age.

## Background

POI is diagnosed in the setting of amenorrhea/oligomenorrhea and serum concentration of FSH > 25 IU/l, monitored two times with at least four weeks apart in women before the age of 40. It has a prevalence of approximately 1% in the general population at the age before 40 years, 0.1% at the age before 30 years [1, 2]. A recent study showed that the pooled prevalence of POI was as high as 3.7% and was found to be higher in in medium and low Human Development Index countries [3]. It can be either primary caused by chromosomal and genetic abnormalities or secondary due to autoimmune disorders, chemotherapy, radiotherapy, infections, and prior pelvic surgery [4]. In recent years, survival after treatments of malignancy continues to improve. Unfortunately, treatment of malignancy such as chemotherapy or radiotherapy is associated with significant ovarian toxicity and has a great risk of POI makes secondary POI becoming more important [5]. A recent review reported pregnancy rates across two randomized controlled trials, two observational studies, and 11 interventional studies ranging from 2.2–14.2%. [6] In populations with POI, egg donation is often the only solution for subsequent infertility, however pregnancy can also occur with hormone replacement therapy (HRT), in-vitro fertilization (IVF), in-vitro maturation (IVM) or with stem cell therapy in 5–10% of cases [7]. Up to now, there is no clinical test can determine the potential for conception in patients with POI. Therefore, diagnosing absolute infertility in women with POI should be prudently, especially in younger women at the age before 30 years. Time and opportunity should be given to these women to have chance to pregnant with their own oocyte rather than donated. We present a case report of cancer survivor patient with POI caused by chemotherapy whose peripheral blood was 46, XY got successful live birth with her own oocyte by appropriate therapy.

## Case presentation

A 31-year-old woman complain of infertility for one and a half years strongly desired to have a child. She has a normal menophania at the age of 14, with menstrual cycle for 28 days and menstrual period lasted 5–7 days. Three years later, at the age of 17, the woman was diagnosed as acute non-lymphocytic leukemia. In order to treat the leukemia, she had received chemotherapy once a month for 11 months followed by bone marrow transplant from a man. One year later, amenorrhea occurred and lasted for 2 years. Then she had oligomenorrhea with the menstrual cycle was once in 6 months for 2 years and once in 1–6 months for 7 years without treatment. Up to now she has been through amenorrhea or oligomenorrhea for 11 years with high serum level of FSH, up to 35.0 IU/L and 53.0 IU/L taken 4 weeks apart, which can be confirmed with POI. Her serum level of AMH was undetectable (< 0.07 ng/mL), luteinizing hormone (LH) was 27.3 IU/l and estradiol (E2) was < 18.35pmol/L. With close to normal secondary sex characteristics, gynecological examination confirmed normal female external genitalia. Transvaginal ultrasound showed near normal size uterus and small ovaries with only one antral follicle. Karyotype analysis showed that the chromosome karyotype of her peripheral blood was 46, XY, which may be diagnosed as Y-chromosome-related DSD. Considered about her past medical history revealed bone marrow transplant from a man, to confirm diagnosis, we run another karyotype analysis using her saliva and the result was 46, XX, which can exclusion DSD. Based on the above characteristics, the woman was diagnosed as iatrogenic POI.

As she had a strong desire to have a child with such poor ovarian function, we decided to use HRT and assisted reproductive technology (ART). Oral contraceptive (Yasmin) was used to reduce gonadotropin hormone until serum FSH down to 12IU/L. Human menopausal gonadotropin (HMG) was used for ovulation induction in the first cycle. One oocyte was retrieved while subsequent IVF was failed. In the second cycle, HMG combined with clomiphene citrate was used, one follicle developed and ruptured in advance. In the third cycle, we used Yasmin combined with HMG, one oocyte was retrieved while IVF was failed. Two days after oocyte retrieval, to strive for an opportunity, we chose to administer luteal phase ovulation induction. Another oocyte was retrieved and fertilized successfully with IVF cultured until day 3 stage embryo. As the implantation window period was missed the embryo was frozen. Gonadotropin-releasing hormone agonist (GnRH-a) and HRT were used for endometrial preparation in FET cycle. 28 days after 3.75 mg leuprorelin injected, HRT started with 3 mg twice/day progynova orally for 7 days and 4 mg twice/day for 7 days till transvaginal ultrasonography displayed the endometrial thickness was 8 mm. Then intramuscular inject progesterone 40 mg twice/day combined with orally dydrogesterone 20 mg twice/day was used to prepare endometrium. After that A 7-cell stage good grading embryo was transferred back into the uterus. A successful pregnancy was confirmed during this cycle and the woman successfully delivered a healthy male newborn.

## Discussion and conclusions

POI used to be described as ‘primary ovarian insufficiency’, ‘premature menopause’ or ‘premature ovarian failure’. As the function of ovarian is volatile and unpredictable, premature ovarian insufficiency is considered to be the preferred term [8]. POI is a heterogeneous disease with possible causes can be divided into genetic, iatrogenic, autoimmune disorders, metabolic, infectious or environmental. However, in the majority of cases of POI, the cause remains a mystery.

A woman with POI and 46 XY karyotype can be easily diagnosed as Y chromosome-related DSD. DSD is defined as congenital conditions in which development of chromosomal, gonadal or anatomical sex is atypical, including a wide spectrum of phenotypes with the clinical characteristics of primary amenorrhea, absent secondary sex characteristics, and abnormal hormone level [9]. In our case the woman was secondary amenorrhea with normal menstrual cycles for about three years instead of primary amenorrhea. After the bone marrow transplant, hematopoietic stem cells have been transplanted, the chromatids of hematopoietic cells will become the donor’s chromosomes, but the chromosomes of the patient’s somatic cells will not be changed. So we can see the result as her chromosome karyotype of her peripheral blood was 46, XY, meanwhile, her chromosome karyotype of her saliva was 46, XX. Though serum level of FSH was extremely high, examinations showed near to external genitalia, internal genitalia and secondary sex characteristics. The tanner staging of her breast was V and her pubic hair was also assessed as a stage V. The transvaginal ultrasound of her pelvic shows normal shape small uterus as 46*46*36mm with a normal 7 mm endometrium. Her ovaries were also small. Her left ovary was 23*14*14 and the right one was 22*17*12mm. Considering about her medical history revealed chemotherapy and bone marrow transplant from a man, we preferred that her POI was caused by oncology treatments and the karyotype of peripheral blood showed 46, XY was caused by BMT. Later the other karyotype analysis of her saliva showed 46, XX confirmed our diagnosis. Huang et al. reported a case of primary amenorrhea after bone marrow transplantation and adjuvant chemotherapy misdiagnosed as DSD [10]. As they noticed that patient presented many characteristics not identical with Y chromosome-related DSD diseases, they recheck her medical record and found her clinical features could be well explained by changed karyotype after BMT. Kruszewska et al. reported a similar case and also mentioned the pitfall of misdiagnosis [11]. These cases remind us a correct diagnosis depends on not only clinical manifestation, physical examination and related adjuvant tests but also the past medical history. Besides, previous studies didn’t mention subsequent therapeutic effects and pregnancy outcomes. Our case provides a possibility for this cohort of patients to get live birth using their own oocyte by appropriate treatments.

Nowadays survival rate of malignant disease has increased significantly thanks to advances in oncology treatment. Nevertheless, it has brought another question about fertility preservation in women. There are about 10% cancers occur in women before the age of 45. Though oncology treatments such as chemotherapy, radiotherapy and bone marrow transplantation can cure over 90% young women with malignant diseases, they can result in POI depending on age, follicular reserve and the regimen of therapies [12]. High doses multiagent regimens such as those required for BMT, just like our case, almost invariably cause immediate POI [13]. According to a population-based analysis, it was observed for all cancer types that cancer survivors are 38% less likely to pregnant compared to general population [14]. Thus, it is essential to develop methods for female fertility preservation, especially in those have to undergoing damaging treatments. The feasibility of ovarian tissue cryopreservation and transportation is nowadays widely documented based on the growing success rate, but still need more research to confirm and optimize the approach [15]. Oocyte vitrification is another effective approach to preserve post pubertal female fertility [16]. Alongside the cryopreservation of gametes, GnRH analogues has also been explored with the aim of protecting ovarian function [17].

However, recent large epidemiological studies showed that the detrimental impact on fertility is less severe if women seek for pregnancy at a younger age [13]. Bramswig et al. reported 467 Hodgkin’s lymphoma survivors who received chemotherapy at the age before 18 showed a significant reduction in frequency of parenthood at the age of 40–44 compared with the general population [18]. The US Childhood Cancer Survivor Study cohort reported 5298 cancer survivors who were treated at the age before 21 showed that survivors who did not report a pregnancy or livebirth before age 30 years had a further reduced likelihood compared with their siblings [19]. Morin’s study demonstrated young women at the aged before 38 with follicular depletion showed no statistically statistical significance in blastulation rates, aneuploidy rates and live birth rates per euploid embryo transfer compared with age-matched women with normal ovarian function [20]. These studies demonstrated age may plays a predominant role in fertility rather than residual ovarian reserve. The quality of oocytes decreases and the aneuploid rate of oocytes increases with age especially at the age over 35. Quality of the oocytes rather than of quantity of the residual primordial follicles would determine the chances of pregnancy. In our case, the younger age as 31 years old when the patient sought for pregnancy laid the foundation for successful pregnancy. Based on above we suggest malignant survivors with POI caused by oncology therapy prepare for pregnancy at younger age.

Up to now, there is no consensus on reliable interventions for women with POI. Though oocyte donation was considered to be the ultimate therapy, there was still a chance for this cohort to conceive with endogenous oocytes spontaneously or by ART. HRT was suggested to alleviate symptoms caused by decreased serum estrogen level in women with POI from the time of diagnosis until the age of natural menopause [21]. Especially in young women, HRT is essential to achieve complete secondary sexual characteristics, and sufficient uterine development for future reproduction. Infertility is also an important problem to deal. Recently Ishizuka et al. suggested to use the regime of ovarian stimulation combined with HRT based on the result that follicle growth was observed in 15.1% of all cycles compared with only 0.2% in patients followed with HRT only [22]. In our case, after fail to obtain embryo by ovarian stimulate protocol of Oral contraceptive combined with HMG and HMG combined with clomiphene citrate in follicular phase, we arranged luteal phase ovulation induction to rescue already developing ovarian follicles. As every follicle seems to be more precious due to ovarian depletion, the more flexible protocol could give another chance for the patient to conceive.

In conclusion, this is a report of a patient with POI and 46, XY karyotype of peripheral blood, which may mislead the diagnosis as DSD, after chemotherapy and BMT. After undergoing HRT, flexible ovarian stimulate protocols and FET, the patient finally obtained successful pregnancy and live birth at the age of 31. It remined that a correct diagnosis depends on not only clinical manifestation, physical examination and related adjuvant tests but also the past medical history. As survival rate of malignant disease increasing significantly, fertility preservation and treatment should be considered for the patient to achieve motherhood especially in younger women.

## Data Availability

The datasets used during the current study are available from the corresponding author on reasonable request.

## Abbreviations

POI: Premature ovarian insufficiency
BMT: bone marrow transplant
FET: frozen-thawed embryo transfer
DSD: disorders of sex development
HRT: hormone replacement therapy
IVF: in-vitro fertilization
IVM: in-vitro maturation
ART: assisted reproductive technology
HMG: Human menopausal gonadotropin
GnRH-a: gonadotropin-releasing hormone agonist
